# Promoting and Risk Factors of Nurses’ Hardiness Levels during the COVID-19 Pandemic: Results from an Italian Cohort

**DOI:** 10.3390/ijerph19031523

**Published:** 2022-01-28

**Authors:** Samuele Baldassini Rodriguez, Yari Bardacci, Khadija El Aoufy, Marco Bazzini, Christian Caruso, Gian Domenico Giusti, Andrea Mezzetti, Stefano Bambi, Andrea Guazzini, Laura Rasero

**Affiliations:** 1 Emergency and Trauma Intensive Care Unit, Careggi University Hospital, 50134 Florence, Italy; samuelebr@hotmail.com (S.B.R.); bardacci.yari@gmail.com (Y.B.); marcobazzini@hotmail.it (M.B.); 2 Department of Experimental and Clinical Medicine, University of Florence, Viale Largo Brambilla, 50134 Florence, Italy; 3 Emergency Medical System—AUSL Toscana Centro, 50134 Florence, Italy; christian.caruso1@gmail.com (C.C.); andrea@mezzetti.it (A.M.); 4 Medicine and Surgery Department, University of Perugia, 06121 Perugia, Italy; giandomenico.giusti@unipg.it; 5 Teaching and Quality Department, Perugia University Hospital, 06121 Perugia, Italy; 6 Department of Health Sciences, University of Florence, 50134 Florence, Italy; stefano.bambi@unifi.it (S.B.); l.rasero@unifi.it (L.R.); 7 Department of Education, Languages, Intercultural Studies, Literatures and Psychology, University of Florence, 50134 Florence, Italy; andrea.guazzini@gmail.com; 8 Center for the Study of Complex Dynamics (CSDC), University of Florence, 50134 Florence, Italy

**Keywords:** resilience, hardiness, stress, anxiety COVID-19, SARS CoV2, nursing, nurses, critical care, healthcare workers

## Abstract

Aim: Few studies in the literature specifically address the hardiness of nurses during the COVID-19 pandemic. Thus, the primary aim of this study was to assess the impact of COVID-19 on the hardiness levels in an Italian cohort of nurses. The secondary aims were to assess the level of hardiness in nurses directly caring for patients with COVID-19 and to verify the presence of related risk and promoting factors. Methods: A descriptive and explorative study was performed through an online survey from March to July 2020. The survey was composed of a multiple answer questionnaire with open, closed, and semi-closed-ended questions. Hardiness and anxiety were assessed using two psychometric instruments: the Dispositional Resilience Scale (DRS-15) and State-Trait Anxiety Inventory (STAI-Y). Results: A total of 1250 nurses completed the questionnaire entirely (92.3% of respondents). The average length of service was 17.8 ± 11.5 years. A decrease in the hardiness was recorded after the first wave of COVID-19 if compared to the baseline (mean Δ DRS-15 total = 1.3 ± 5.0), whereas in the subsample of nurses caring for COVID-19 patients, the total hardiness level decreased more consistently (mean Δ DRS Total = 1.9 + 5.3). Multivariate analysis showed that high levels of anxiety were risk factors for reducing hardiness. In contrast, anxiety, when associated with a greater length of service, was a promoting factor for the increase in hardiness. Conclusions: The correlation between anxiety and years of length of service appears to be pivotal. Future research should focus on the role of anxiety to establish its actual role as a predictor of hardiness.

## 1. Introduction

### 1.1. Background

The COVID-19 pandemic has changed individuals’ lives all over the world during the past two years. Beyond the tragic consequences in terms of number of deaths, and the effects on the physical quality of life of survivors affected by “Long-COVID” [1], the COVID-19 pandemic has also considerably impacted the psychological and psychopathological status of populations, in terms of distress, anxiety, depression, and post-traumatic stress disorder (PTSD) [2]. Stress-related disorders, depression, and anxiety have also been commonly reported among healthcare workers [3,4,5]. The main psychological challenges faced by healthcare workers are the scare of infection and, in general, an “unknown” condition [6]. Resilience has a pivotal role in improving and enhancing the workers’ response to crisis, and ultimately the healthcare systems [7,8]. Indeed, resilience indicators and self-efficacy have been shown to be protective factors for mental health outcomes in healthcare professionals during epidemic outbreaks, and especially during the COVID-19 pandemic [5,9,10].

Resilience is defined as “the ability to react to stress in a healthy way such that goals are achieved at minimal psychological and physical cost” [7]; this concept involves human responses to the adversity that leads to thriving, rather than simply surviving [11]. Thus, persons with adequate resilience levels can rapidly overcome difficulties and become stronger than before the events, preventing negative mental health issues [12].

Numerous variables affect the development of adequate resilience levels, and these variables are also related to the individuals: effective coping strategies, perception of positivity in life, giving a meaning and sense of positive growth to stress and traumatic events, spirituality, and dispositional optimism. Ego resiliency and hardiness are significant psychological tracts in individuals’ resilience [13]. As reported by Bartone, “Hardiness, also named ‘dispositional resilience’ is a personality style associated with resilience, good health, and performance under stressful conditions” [14,15], which can predict the adaptation of individuals to stressful and traumatic events [16]. The concept of hardiness was first elaborated by Kobasa during the 1980s, referring to a personality style that protects against the psychophysical symptoms arising from work or life stressful events [17].

The concept of resilience is depicted by different disciplines through three main themes: hardiness, which increases the ability to use resources; regulatory flexibility, which promotes positive functioning; and challenges, which improve the capability to recover [18]. Hardiness can also be viewed as an antecedent of resilience; indeed, it is considered a means to achieve resilience, thus improving the protection from the impact of high levels of stress [18]. According to existential theory, hardiness is composed of attitudes or beliefs that constitute courage and motivation to deal with stressful events [19]. Therefore, hardiness represents a personality trait predicting health, performance, and behavioral outcomes [19]. Its construct comprises three dimensions: commitment (versus alienation), control (versus powerlessness), and challenge (versus threat) [19].

Commitment refers to the levels of involvement and the meaning attributed to life events; control is related to the individual’s perception of affecting the events of their own life; and challenge refers to the willingness to live through changes, and the sense of positive growth resulting from good and bad life experiences [20]. Eschleman et al., in their meta-analysis, found that hardiness was positively related to personality protecting factors against stress, and to active coping, performance, and social support. Moreover, a negative relationship between hardiness and personality characteristics, which increase stress and diminish coping strategies, was confirmed [21]. Finally, low levels of hardiness are also related to higher scores of psychophysical complaints [22].

Accordingly, hardiness showed statistically significant negative correlations with scores of burnout cynicism, inefficacy, and exhaustion subscales among Japanese psychiatric hospital nurses [23].

Nonetheless, few papers about the measures of hardiness in nurses and physicians during epidemic and pandemic outbreaks are available. Park et al. (2018) explored hardiness levels in nurses during the MERS-CoV outbreak, showing that it affected mental health both directly and indirectly [24]. Recently, two Italian studies addressed the effects of the COVID-19 pandemic on healthcare personnel during the first wave [25,26]. The results showed that nurses and physicians experienced higher levels of emergency stress than emergency workers, and that coping strategies and hardiness were protective factors for stress, reducing its effect on secondary trauma [25]. According to Vagni et al., commitment was related to stress, arousal, and intrusion, whereas control exerted a protective function [26].

The scientific literature focusing on hardiness and resilience is increasingly developing; however, few studies have specifically addressed the hardiness of nurses during the COVID-19 pandemic. For this reason, an observational study was designed to estimate the level of hardiness in nurses directly involved (or not) in the care of COVID-19 patients, and to explore the variables that can affect this specific trait of personality. The present paper reports the descriptive and exploratory analysis of hardiness derived from data collection performed during the AIR-COVID-19 study, an observational study aimed to evaluate anxiety, insomnia, and dispositional resilience levels in healthcare workers during the COVID-19 pandemic.

### 1.2. Aims of the Study

The primary aim of the present study was to assess the impact of COVID-19 on hardiness in an Italian cohort of nurses. The secondary aims were to assess the level of hardiness in nurses directly caring for patients with COVID-19, and identify the presence of related risk and promoting factors.

## 2. Materials and Methods

### 2.1. Study Design

A descriptive- explorative study was performed by developing an online survey during the first wave of the COVID-19 pandemic (from March to July 2020).

### 2.2. Participants

The original sample of the AIR-COVID-19 study was composed of all the healthcare professionals working in and out of hospital settings directly involved in the care of COVID-19 patients during the first surge of the pandemic emergency, and those who cared for non-COVID-19 patients. However, in this study only nurses were included.

Inclusion criteria were the following: all national healthcare system workers with an unlimited or fixed-term employment contract and voluntary acceptance of the informed consent for study participation. No exclusion criteria were applied, except for the lack of consent to participation in the study. No sample size was calculated because of the descriptive design of this study, with the aim to include as large a number of participants as possible.

### 2.3. Methods

A web survey was implemented through the Survey Monkey online platform, offered by the Italian Association of Critical Care Nurses (Aniarti). The link to the questionnaire was made available through healthcare professional associations’ pages on the web and social networks. The survey period started in May 2020, immediately after the first wave of the pandemic, and continued for 60 days. To complete the questionnaires, a mean time of 10–12 min was required, and the participants could leave the study at any moment.

### 2.4. Measures

The survey was composed of a multiple answer questionnaire with open and closed questions, and semi-closed-ended questions. The closed questions included various typologies of answers: multiple, dichotomous, or ratings (Likert-type scale). The first section of the questionnaire collected demographic data. Some items were designed to collect data about participants’ healthcare settings, their involvement in COVID-19 patients’ care, the change in workplace due to the pandemic emergency, and the distance from home to their current workplace. The second section of the survey was composed of the Italian versions of two psychometric instruments: the Dispositional Resilience Scale (DRS-15) [13] and the State-Trait Anxiety Inventory (STAI-Y) [27]. Hardiness was assessed by the Dispositional Resilience Scale (DRS-15), which is a valid, reliable, and brief psychometric instrument for the self-assessment of hardiness. This scale measures the resiliency levels in terms of psychological resistance, which is a general functioning style including cognitive, emotive, and behavioral features.

DRS-15 explores three dimensions of resilience (subscales): “Commitment”, “Control”, and “Challenge” [13]. The original DRS was composed of 45 items and three subscales (communication, challenge, and control); it was then reduced to 30 items, and finally to 15. DRS-15 included three subscales (Commitment, Challenge, and Control) with acceptable psychometric properties (Cronbach a of 0.84 and well-established criterion validity) [14]. In this study the validated Italian version with 15 items was administered, and showed good reliability and predictive validity levels. Each item asks the participants to state the level of truth about a single affirmation on a 4-point Likert scale (from “1—not at all true” to “4—completely true”).

Furthermore, a good test–retest reliability was found (0.78), even if the subscale “control” showed a lesser value (0.58) than the “commitment” and “challenge” subscales (0.75 and 0.81, respectively). Construct validity was confirmed for two factors, except for the challenge factor [14]. DRS-15 is also available in Chinese and Portuguese versions, which show good levels of internal consistency, stability, and construct and criterion validity [15,28]. Picardi et al. (2012) undertook the cross-cultural adaptation of DRS-15 for the Italian language. The Italian version of DRS-15 showed good levels of reliability and stability (Cronbach a of 0.73; Intra-Class Correlation of 0.75 between two administrations after a time interval of one month), and evidence of construct and criterion validity [13].Anxiety was assessed by the State Trait Anxiety Inventory (STAI-Y), which is a 40-item questionnaire used to measure both state and trait anxiety [29]. In STAI-Y1 (items 1–20), the intensity of feelings “in this moment” is assessed, whereas in STAI-Y2 (items 21–40) the focus is on the frequency of feelings “in general”. Both parts of the questionnaire use a 1–4 Likert rating scale [27]. The detection of both state and trait anxiety was measured with STAI-Y. In the present study, we considered only the level of state anxiety for two reasons: it is more sensitive when compared to trait anxiety and it is actually highly related to trait anxiety (0.8) [30].

### 2.5. Ethical Considerations

No ethical approval was needed according to local ethical committee (Tuscany Regional Ethical Committee) policy because no patient was involved in this study. The study protocol was drafted according to the Good Clinical Practice (GCP) and was the study was conducted according to the principles of Helsinki Declarations. The researchers performed the study following the guidelines contained in the new national Privacy Body of Law (Italian laws numbers 196/2003 and 101/2018). All participants’ data were collected and anonymity was maintained. Moreover, data were analyzed through aggregated forms. The results were transferred via an *xls* file with a password known only by the researchers. The personal information of the respondents was made anonymous through the assignment of an individual sequential code number.

## 3. Data Analysis

The data analysis procedure was subdivided into four phases. In the first step, we preprocessed, codified, and cleaned the datasets coming from the survey, and discretized and changed the metrics of the observables whenever the balancing of the conditions did not satisfy the prerequisite for the subsequent inferential analysis. In the second step, analyses of frequencies, central tendencies, and dispersion indicators were carried out using the IBM Statistical Package for Social Sciences (SPSS 27.0) [31]. Then, before proceeding with inferential analyses, we checked that the data respected the analyses’ assumptions and, thus, we verified the normality of the distribution of the continuous variables. Thus, we assessed that the asymmetry and kurtosis values fell in the interval between −1 and +1, and that a sufficient balance and size of the subsamples was achieved considering the discrete dimensions. In the third step, we investigated the univariate relations between the selected observables, adopting the Pearson *r* correlation to compare continuous variables, and the repeated measures ANOVA to evaluate the impact of dichotomous observables on continuous ones and, in particular, the effects of time (i.e., pre- and post-pandemic first-wave effect) on hardiness. Finally, in the fourth step we investigated the combined effects of the hardiness risk and promoting factors involved in the study by adopting a logistic regression model. We discretized the perceived change in hardiness into two levels (i.e., indicating with 0 when it decreased due to COVID-19, and with 1 when it was maintained or increased), in order to evaluate the impact of continuous and discrete variables, and their combined effects.

## 4. Results

### 4.1. Descriptive Analysis of the Whole Sample (All the Nurses Included in the Study)

The original respondents of AIR-COVID-19 were 1693 healthcare providers, of which 1354 were nurses. In this study, 1250 nurses were included as they filled out the questionnaire correctly, thus representing 92.3% (1250/1354) of respondents (of which 81.4%—1017/1250 women and 18.7%—234/1250 men). The average age of respondents was 42.3 ± 10.6 years (M ± SD; range 21–66 years).

Respondents reported an average length of service of 17.8 ± 11.5 years (M ± SD; range 0.5–43 years), and 4.7% had less than one working year. This sample seems to be representative of the general population of nurses, given that official Italian Health Ministry statistics for 2018 reported a mean age of 47.7 years and an average length of service of 19 years [32].

Regarding the geographical origin, 46.3% were from the North, 47.8% from Central Italy, and 5.8% were recruited from health workers in Southern Italy.

Before the pandemic, 33% (412/1250) of the respondents worked in the medical or surgical units, 28.9% (361/1250) in intensive care units, 15.7% (196/1250) in Emergency Departments (EDs), 5.9% (74/1250) in an operating room, 2.1% (26/1250) in an infectious disease unit, 3% (38/1250) in the emergency medical system, 9% (113/1250) in the territorial services, and the remaining 2.5% (31/1250) did not specify their work setting (Table 1). Then, as a consequence of the COVID-19 pandemic, change in the work setting was asked: 28.2% (352/1250) of the respondents reported having changed the unit they were belonged to, and 81.8% (288/352) were transferred to a COVID-19 unit. On average, transferees reported a positive satisfaction rate of 77.7%: 26.5% were very satisfied, 23% were quite satisfied, and 28.2% were satisfied, whereas 22.4% were dissatisfied (13.1%) or not at all satisfied (9.3%) with the transfer.

A total of 412 nurses of 1250 (33%) reported caring for COVID-19 patients, whereas the remaining 67% (838/1250) did not. In addition, the perceptions about being adequately provided with personal protective equipment (PPE) were investigated: 4.3% (54/1250) stated they did not receive PPE, 43.6% (545/1250) received a partial or incomplete endowment, and 52.2% (652/1250) were satisfied about the PPE received.

### 4.2. Descriptive Analysis of the Nurses Directly Involved in Caring for Patients with COVID-19

The subsample of nurses who claimed to care for COVID-19 patients was 76.9% female (317/412), with an average age and length of service of 41 ± 11.3 years (M ± SD) and 16.7 ± 11.3 years (M ± SD), respectively. This subsample appears to be adequately representative of the Centre (51.2%; N = 211) and North (46.6%; N = 192) of Italy, where only 1.9% (8) came from the South. Moreover, 28.4% (117/412) of this subsample reported to have changed their initial work setting due to the pandemic; Table 2 shows the actual setting during the pandemic.

### 4.3. Descriptive Analysis of the Hardiness

The hardiness level of the nurses was assessed for the whole sample, and both the subsamples involved in the care of COVID-19 patients and not-COVID-19 patients. Descriptive analyses are shown in Table 3 and Table 4. Results show a decrease in the hardiness levels assessed after the first wave of COVID-19 compared to the baseline (DRS-15 total delta 1.3 ± 5.0 M ± SD), whereas in the subsample of nurses caring for COVID-19 patients the total hardiness level decreased more consistently compared to the whole sample (delta DRS total of 1.9 + 5.3 M ± SD).

### 4.4. Descriptive Analysis of the Anxiety Assessed by the STAI-Y

The 1250 respondents reported an anxiety value of 46.9 ± 12.4 (M ± SD), whereas the subsample of nurses who cared for COVID-19 patients reported a value of 48.7 ± 12.7 (M ± SD).

### 4.5. Correlations between Nurses Caring for COVID-19 Patients and the Level of Hardiness

**Figure 1:**
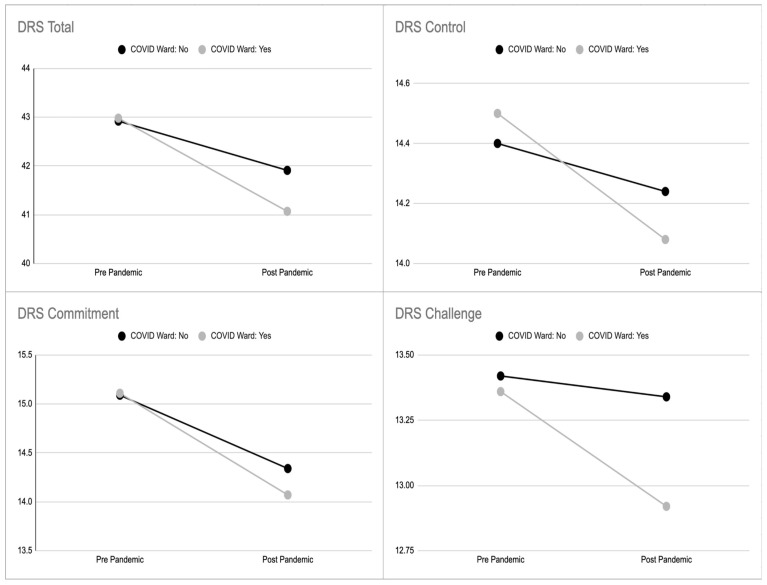
Average level of hardiness (total, commitment, control, and challenge) in the two subsamples of nurses taking care of COVID-19 patients and those who did not, with respect to time (i.e., pre- and post-pandemic first-wave effect).

An inferential analysis was performed to assess the impact of caring for COVID-19 patients on hardiness, compared to nurses not involved with COVID-19 patients. As reported in Table 5, Table 6, Table 7 and Table 8, all the subscales of DRS-15 showed a statistically significant difference. In particular, the ANOVA analyses always reported a significant effect of time (i.e., pandemic) on the whole sample. Nevertheless, participants who were moved after the start of the pandemic in a COVID-19 ward reported significantly lower scores. Figure 1 clearly shows that the slopes of the lines connecting pre- and post-pandemic scores appear to be lower for the COVID-19 ward subsample, disregarding their starting scores.

### 4.6. Correlations between Sociodemographic Parameters, Geographic Area, Other Factors and Levels of Hardiness in the Group of the Nurses Taking Caring for COVID-19 Patients 

#### 4.6.1. Sociodemographic Factors

No significant differences were found in either the final DRS values, the pre and post variation in relation to gender, age, seniority of service, or department change, or in the relation to the delta of hardiness. No linear relationship was found; however, non-linear relationships are discussed in the following paragraphs.

#### 4.6.2. Personal Protective Equipment (PPE)

An inferential analysis was performed to assess the impact of the perception of adequate (or not) provision of PPE on the hardiness level of nurses caring for COVID-19 patients. As reported in Table 9, Table 10, Table 11 and Table 12, all the subscales of DRS-15 showed a statistically significant difference. 

**Figure 2:**
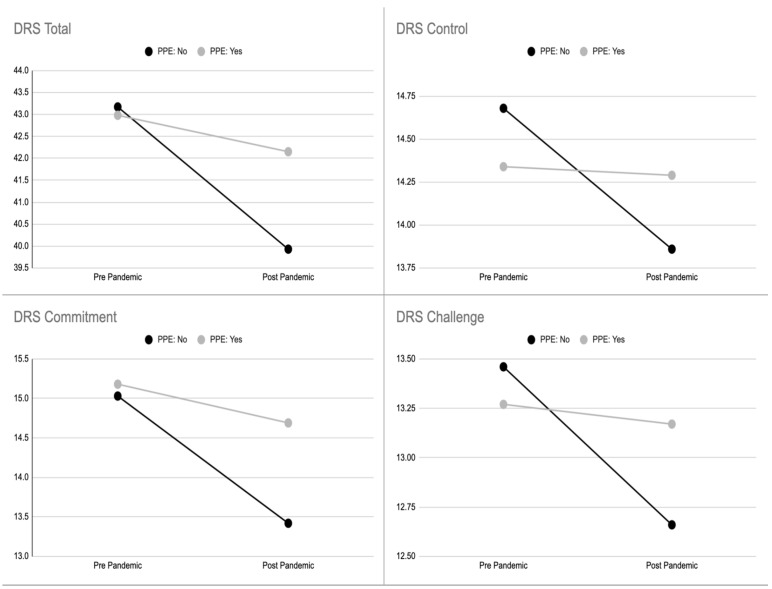
Hardiness levels (total, commitment, control, and challenge) of nurses taking care of COVID-19 patients, shown in the two subsamples of those adequately provided with PPE and those who were not.

Figure 2 clearly shows how the slopes of the lines connecting pre- and post-pandemic scores appear to be lower according to the subsample of nurses claiming inadequate PPE (NO), despite their starting scores.

#### 4.6.3. Satisfaction Score According to Changes in Work Setting 

Changing the ward or department of allocation showed a significant association with the four dimensions of hardiness in the group of nurses caring for COVID-19 patients, as reported in Table 13. The analysis of the correlation between transfer (department/ward reallocation) satisfaction and post-transfer dispositional resilience shows that there was a common variance of between 10% (DRS challenge) and 25% (DRS commitment). This implies a positive association between the satisfaction about the transfer and the hardiness (the higher the satisfaction, the higher the hardiness). In particular, as satisfaction increased, the positive hardiness change increased, and vice versa. The variance explained was comparable to the that related to post-resilience, with the exception of DRS control, in which it appeared to be greater (15% vs. 22%).

#### 4.6.4. Geographical Area 

**Figure 3:**
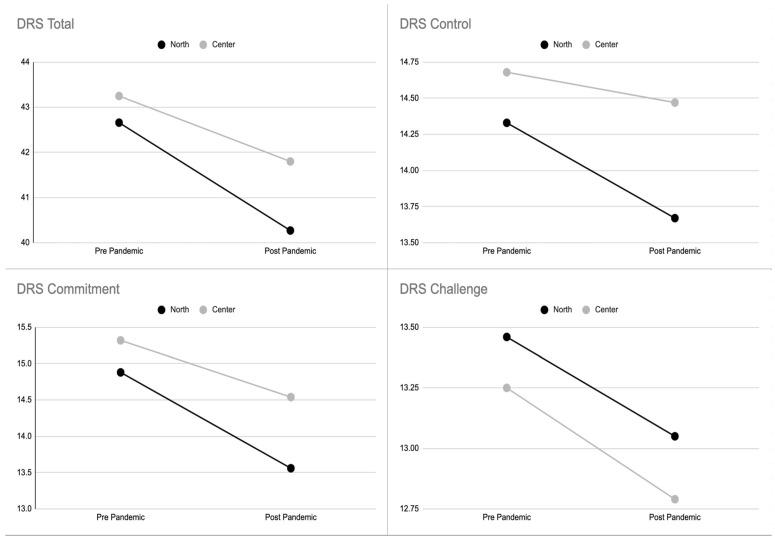
Hardiness levels (total, commitment, control, and challenge) in the two subsamples of nurses from Northern Italy and those from Central Italy.

The geographical area of Southern Italy was excluded from the analysis given the few respondents (*n* = 8). Central and Northern Italy were included in the statistical analysis (Table 14, Table 15, Table 16 and Table 17). The analysis showed higher levels of hardiness with regard to commitment, control, and the total scale for the nurses caring for COVID-19 patients from Central Italy, with the exception of the challenge subscale (Figure 3), compared to the nurses from Northern Italy. However, there were significant differences only for commitment and control subscales when combined with the geographical area.

#### 4.6.5. State Anxiety

State anxiety was considered to be a possible mediator of hardiness. A significant and negative correlation between levels of state anxiety and all dimensions of resilience was found (Table 8). The total post-COVID-19 DRS value seems to have a common variance with the state anxiety of 38%, (17% for control, 24% for challenge, and 29% for commitment). In particular, the increase in anxiety levels corresponded to a reduction in all the dimensions of dispositional resilience. Regarding the pre-/post-DRS difference, it is appreciated that a greater improvement in dispositional resilience (positive delta) corresponded to a reduction in state anxiety, whereas a worsening in dispositional resilience (negative delta) corresponded to an increase in anxiety. In particular, the association between anxiety and resilience variation appeared to be 11%, 14%, 20%, and 23% for the challenge, control, commitment, and total scales, respectively (Table 18). 

### 4.7. Predictors of the Multivariate Analysis for Hardiness Level in Nurses Taking Care of COVID-19 Patients

Independent determinants of hardiness in Italian nurses directly involved in caring for patients with COVID-19 were evaluated in relation to the total score and subscales of DRS-15, together with anxiety level, PPE availability, satisfaction of work setting reallocation, and sociodemographic factors. Each item of the DRS scale (Delta Total, D-Challenge, D-Commitment, D-Control) was investigated for risk or promoting factors/mediators through a logistic regression. In order to assess possible interactions between the dimensions of interest and the variation in dispositional resilience due to the COVID-19 pandemic, the delta DRS size was discretized by shortening the cases of increased or maintained dispositional resilience, and separating them from the cases of reduced DRS levels.

#### 4.7.1. Predictors for Hardiness Level in Nurses Taking Care of COVID-19 Patients

**Figure 4:**
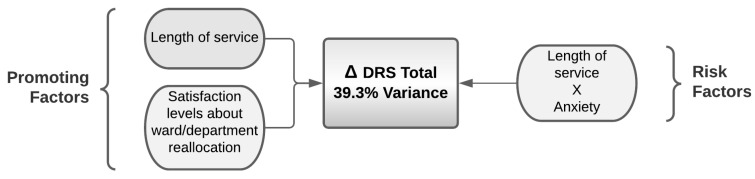
Promoting and risk factors for the variance in the hardiness assessed by DRS total resulting from the logistic regression.

The logistic regression reported in Table 19 shows the complexity of the relation between the predictive variables and the variation in the total dispositional resilience (post–pre); 39% of this variance can be explained by the model. Indeed, length of service seemed to be a protective factor showing a significant relationship with maintained or improved hardiness scores. Moreover, the evaluation of the transfer had a specific role, and assumed the greatest weight in this model. Finally, an interaction between length of service and the effect of state anxiety is highlighted; length of service is normally a protection factor for hardiness, although in this model it represents a risk factor when combined with state anxiety. In other words, anxious nurses with a long length of service had a reduced total hardiness score compared to people with a shorter length of service (Figure 4).

#### 4.7.2. Predictors for Commitment Levels in Nurses Caring for COVID-19 Patients

**Figure 5:**
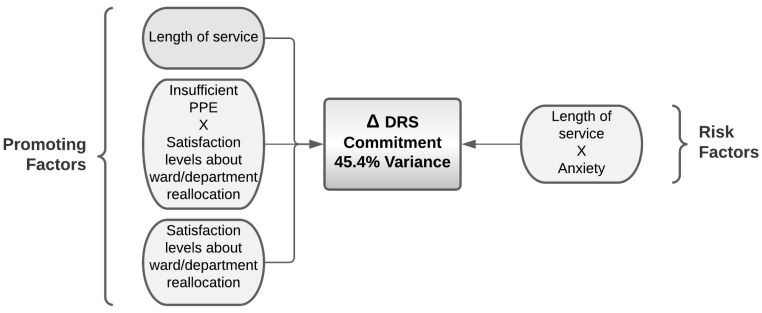
Promoting and risk factors for the variance in the hardiness assessed by DRS commitment resulting from the logistic regression.

Regarding the commitment score, the best generated model explains 45% of its variance; higher length of service is confirmed as a protective factor, and the satisfaction levels regarding ward/department reallocation and the interaction between length of service and state anxiety are also protective factors. When the PPE were considered to be absent, higher scores of satisfaction levels regarding ward/department reallocation were associated with higher commitment, compared to the case when PPE was considered to be well provided (Table 20 and Figure 5).

#### 4.7.3. Predictors for Control Levels in Nurses Caring for COVID-19 Patients

**Figure 6:**
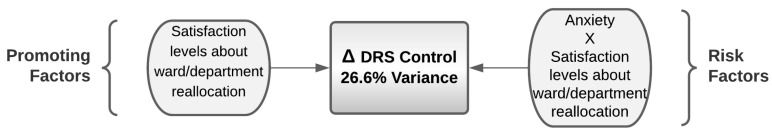
Promoting and risk factors for the variance in the hardiness assessed by DRS control resulting from the logistic regression.

Regarding the control score, the best model predicted 27% of its variance; satisfaction level about ward/department reallocation was found to represent a promoting factor, whereas, when combined with high anxiety levels, it is a risk factor for control levels. Thus, higher anxiety scores showed a significant impact on the positive satisfaction levels about ward/department reallocation, which consequently decreased (Table 21 and Figure 6).

#### 4.7.4. Predictors for Challenge Levels in Nurses Caring for COVID-19 Patients

**Figure 7:**
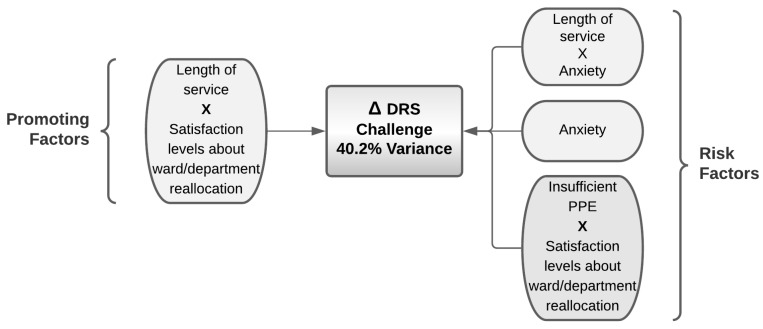
Promoting and risk factors for the variance in the hardiness assessed by DRS challenge resulting from the logistic regression.

Regarding the challenge score, the model predicted 40% of its variance (Table 22 and Figure 7); here, anxiety levels were found to be a risk factor both alone and in interaction with length of service. The lower impact on challenge scores, despite the level of satisfaction about ward/department reallocation, was explained by the PPE being rated as absent. In addition, an interaction between length of service and the levels of satisfaction about ward/department reallocation represented a promoting factor for hardiness.

## 5. Discussion

Italy was the first European country that was required to manage the COVID-19 emergency, and implemented a large number of social, economic, and healthcare changes [33]. Nurses’ managers, as a consequence of hospitals’ re-organizations, had to deal with a contingent of nurses who were newly hired or moved into more complex clinical settings, and gap in critical care competencies had to be quickly filled [34,35]. Similar circumstances also occurred during the reconversion of general wards into COVID-19 acute care wards. Finally, nursing workloads during the COVID-19 pandemic increased significantly [36,37] with long work-shifts and inadequate time to rest [38]. The high work pressure and uncertainty about the risks of COVID-19 increased nurses’ anxiety, depression, post-traumatic stress disorder [39], emotional exhaustion [38], and burnout [40] rates.

Noting these implications for nurses, we designed a study aiming specifically to assess the hardiness level, its dimensions, and their predictors. As expected, our results showed a significant difference in the hardiness’ level for nurses caring directly for COVID-19 patients (delta 1.0 ± 4.8 M ± SD) compared to those not involved directly with COVID-19 patients (delta 1.9 ± 5.3 M ± SD) (Table 4).

Despite the amount of literature, to date, few studies have been published about psychological hardiness levels of nurses and other healthcare professionals during the COVID-19 pandemic. In Italy, Vagni et al. studied the levels of hardiness and stress in 140 healthcare workers and 96 emergency workers (ambulance personnel, firefighters, police, and Civil Protection) involved in the COVID-19 pandemic, using DRS-15 [26]. They did not find statistically significant differences between the mean scores of the commitment subscale (9.81 ± 1.88, versus 10.49 ± 1.97), and the control subscale (10.39 ± 1.96, versus 10.65 ± 2.67), whereas a slight difference emerged between the scores of the challenge subscale of healthcare workers and emergency workers (7.96 ± 2.18 and 7.92 ± 1.90, respectively; *p* < 0.01) [27]. Moreover, there were no significant differences in the mean scores of the DRS-15 subscales between those who directly cared for COVID-19 patients and those who did not (control 10.47 ± 1.98 versus 10.53 ± 2.24; challenge 7.89 ± 2.15 versus 8.01 ± 1.95), with the exception of the commitment subscale (9.87 ± 1.98 versus 10.38 ± 1.86, *p* < 0.05) [27].

Overall, healthcare workers showed moderate to high levels of resilience during the COVID-19 pandemic [10]. In particular, the resilience levels among healthcare professionals caring directly for COVID-19 patients reported by a literature review performed on 32 studies were found to lie in a range of moderate scores [9]. Accordingly, another study, performed by Jose et al. (2020), reported that 47.5% of 120 nurses in an Indian emergency department during the COVID-19 pandemic showed moderate to high levels of hardiness [40], assessed with the subscale “Hardiness” (8 items) of the Connor–Davidson Resilience Scale-25 [40]. Indeed, in our sample, statistically significant differences in hardiness levels (as a forecaster of the resilience), between nurses caring for COVID-19 patients and those who did not, were also confirmed for the subcategories of commitment, challenge, and control. 

Importantly, hardiness is not an intrinsic personal trait, but it can be learned and internalized [41], as demonstrated by many studies on diverse populations (nurses, other healthcare workers, sport coaches, military personnel, and undergraduate students) [42].

Regarding the geographical area, working in the North of Italy was associated with lower scores of hardiness compared to the Central Italy; indeed, the earliest phase of the COVID-19 pandemic mainly affected the North of Italy, with tragic consequences for the healthcare system and a higher number of deaths [43]. Southern Italy was not included in the analysis due to the small number of participants. Lasalvia et al. reported in their study that the psychological impact of the COVID-19 pandemic on healthcare staff working in a highly burdened geographical area of north-east Italy was relevant and, to some extent, greater than that reported in China [44].

Furthermore, the organizational settings changed during the pandemic and forced the healthcare systems to adapt immediately with new COVID-19 units and COVID-19 ICUs, to deal promptly with the healthcare emergency. Considering the change in clinical setting, it would be expected that the assignment of nurses to COVID-19 units had a great impact on the hardiness level. However, our results did not completely confirm this hypothesis, as shown by the small delta values (Table 3 and Table 4). Despite this, the linear relationship between the satisfaction regarding the work setting reallocation showed a significant and positive association with the hardiness levels, with a variance between 10% and 25%, and a particular impact on the measures of commitment and control. A positive perception of the work setting reallocation, here, is strictly linked to a higher hardiness level. Thus, taking into account the perception of nurses, and health professionals in general, may have a positive impact on hardiness.

Regarding the supply of PPE, an interesting difference between the perception of being provided with adequate PPE, or not, was observed. Thus, nurses who claimed to have received adequate PPE according to the government standards of provisions reported a higher level of total hardiness and commitment assessed during the first wave of the COVID-19 pandemic. It is largely reported in the literature that PPE made the difference in individuals’ perception, and that stress and anxiety increased when people were not adequately protected [45,46]. Maiorano et al. found that caring for COVID-19 patients, female sex, unforeseen events, and lack of PPE were found to be risk factors for emergency workers’ stress [25].

A recent study performed on Iranian nurses involved in the care of COVID-19 patients showed moderate levels of negative correlations between hardiness and stress, and a positive correlation with mental health (Pearson correlation coefficient −0.581, *p* < 0.05 and 0.474, *p* < 0.01, respectively) [47]. Similar results were found by Park et al. on 187 nurses in a South Korean Hospital during the MERS virus outbreak in 2015 (Pearson’s coefficients −0.401, *p* < 0.001 and 0.439, *p* < 0.001, respectively) [24].

The nurses included in our study showed a level of state anxiety that was 90% higher than that of the general population [48]. The state anxiety level significantly affected all the spheres of hardiness, i.e., both the delta and the assessment after the first pandemic wave. Hardiness and anxiety have a relevant role in the well-being of healthcare providers and ultimately, therefore, in the quality of care provided. 

Numerous studies have focused on risk and protective factors to address strategies for developing interventions to reduce and strengthen these psychological issues, respectively [8,22]. Resilience resources are factors that are able to counterbalance stressful events or risk factors for stress, and can be found at multiple levels: personality, family, community, and society [13]. However, the association of anxiety with length of service seems to have a negative impact on hardiness level; thus, anxiety has an indirect negative impact on hardiness level. In particular, as the level of anxiety increased, the dispositional resilience of subjects with a longer length of service was reduced. Our results showed a different level of hardiness before and during the pandemic, which has not changed significantly. In addition, nurses with a longer length of service and no anxiety experienced higher levels of hardiness compared with colleagues with a shorter length of service, whereas nurses with a longer length of service, combined with anxiety, experienced lower hardiness’ levels.

Given the ambiguity of these results, the role of anxiety was hypothesized to have an interaction with the length of service in affecting the perception of hardiness. According to the results mentioned above, length of service is a protective factor for hardiness, and particularly in regard to commitment and challenge. Nonetheless, the interaction of length of service with higher levels of anxiety is a risk factor that negatively affects all spheres of hardiness.

By comparison, the same length of service, associated with higher levels of satisfaction for work setting reallocation, is a protective factor for challenge, as an antecedent of hardiness. Interestingly, in the sphere of commitment, despite the nurses claiming that they did not receive adequate PPE, the positive evaluation of the unit reallocation allowed it to be a protective factor.

### Limitations and Suggestions of the Study 

Some limitations of the present study must be mentioned. Self-selection bias may have occurred among nurses who decided to complete the questionnaire; the survey was also only disseminated through a critical care nurses’ association (Aniarti).

Moreover, the choice of administering the hardiness scale DRS-15 twice, i.e., asking the participant about their current level of hardiness and their hardiness before the pandemic, may have resulted in recall bias. However, the study was designed during the pandemic and it was not possible to assess this variable previously.

Additionally, the national representativeness of the sample is lacking because it did not include Southern Italy. There were few participants from this geographical area, even though they comprised the population of nurses less affected by COVID-19 during the first wave. Finally, despite the large number of participants, we recognize that a huge number of surveys were available during the same period, affecting the willingness of healthcare professionals to participate in our survey.

## 6. Conclusions

Our results showed statistically significant differences in hardiness levels between nurses directly caring for COVID-19 patients and those who did not. DRS variance was found to be significant and higher in the first group of nurses.

Based on this study, the role of anxiety levels needs to be closely reconsidered because it may seem to be contradictory. Indeed, when dealing with the predictors of hardiness, anxiety has the role of a hardiness proxy. Promoting factors for hardiness in nurses involved in the care of COVID-19 patients were length of service, positive assessment of department reallocation, and, surprisingly, inadequate PPE when considering a positive assessment of department reallocation. In contrast, the risk factors, which reduced levels of hardiness, were anxiety alone and associated with length of service, a negative assessment of department reallocation, and insufficient PPE when associated with a negative assessment of department reallocation.

Given the pivotal role of hardiness on the quality of care and on the individual health of nurses globally, future research should address this topic in order to establish the actual role of the predictors of hardiness, and to confirm or disagree with our findings. The variation in hardiness levels suggests that this personal trait may be affected by contingencies. Indeed, predictive factors in terms of risk or promotion need to be addressed using a more structured study design.

## Figures and Tables

**Table 1:** Reallocation of nurses included in the study due to the COVID-19 pandemic.

Work Setting	Before COVID-19 N. (%)	Reallocation Due to COVID-19 N. (%)
Intensive care unit	361 (28.9%)	71 (5.7%)
Intensive care unit COVID	0%	430 (34.4%)
Medical-surgical Unit	412 (33%)	251 (20.1%)
Medical-surgical Unit COVID	0%	59 (4.7%)
Emergency Department	196 (15.7%)	195 (15.6%)
Infectious Diseases Unit	26 (2.1%)	57 (4.6%)
Emergency Medical System	38 (3%)	23 (1.8%)
Operating Rooms	74 (5.9%)	20 (1.6%)
Territorial Services	113 (9%)	126 (10.1%)
Other	31 (2.5%)	19 (1.5%)

**Table 2:** Work setting of nurses directly taking care of COVID-19 patients during the pandemic.

Work Setting of Nurses Directly Taking Care of COVID-19 Patients N. (%)	
Intensive care unit	5 (1.2%)
Intensive care unit COVID	181 (43.9%)
Medical-surgical Unit	26 (6.3%)
Medical-surgical Unit COVID	20 (4.9%)
Emergency Department	84 (20.4%)
Infectious Diseases Unit	29 (7%)
Emergency Medical System	20 (4.9%)
Operating Rooms	4 (1%)
Territorial Services	42 (10.2%)
Other	1 (0.2%)

**Table 3:** DRS total, commitment, control, and challenge scores reported as mean and standard deviation for the entire sample.

Phase Observable	PRE (M ± SD)	POST (M ± SD)	Δ (M ± SD)
	Entire Sample *n* = 1250	Entire Sample *n* = 1250	Entire Sample *n* = 1250
DRS: Total score	27.9 ± 5.5	26.6 ± 6.7	1.3 ± 5.0
DRS: Commitment	10.1 ± 2.4	9.2 ± 3.0	0.8 ± 2.3
DRS: Control	9.4 ± 2.2	9.2 ± 2.4	0.2 ± 1.9
DRS: Challenge	8.4 ± 3.0	8.2 ± 3.2	0.2 ± 1.9

Legend: M: Mean; SD: Standard Deviation; DRS: Dispositional Resilience Scale; PRE: Pre-COVID-19 period; POST: Post-COVID-19 period; Δ: difference between post- and pre-COVID-19 periods.

**Table 4:** DRS total, commitment, control and challenge scores reported as mean (M) and standard deviation (SD) for the nurses taking care of COVID-19 patients and those who did not.

Phase Observable	PRE (M ± SD)		POST (M ± SD)		Δ (M ± SD)	
	NO COVID-19 (*n* = 838)	COVID-19 Units (*n* = 412)	NO COVID-19 (*n* = 838)	COVID-19 Units (*n* = 412)	NO COVID-19 (*n* = 838)	COVID-19 Units (*n* = 412)
DRS: Total score	27.9 ± 5.6	28.0 ± 5.2	26.9 ± 6.6	26.1 ± 6.9	1.0 ± 4.8	1.9 ± 5.3
DRS: Commitment	10.1 ± 2.4	10.1 ± 2.3	9.3 ± 3.0	9.1 ± 3.1	0.7 ± 2.2	1.0 ± 2.4
DRS: Control	9.4 ± 2.2	9.5 ± 2.1	9.2 ± 2.4	9.1 ± 2.4	0.2 ± 1.9	0.4 ± 1.9
DRS: Challenge	8.4 ± 3.0	8.4 ± 3.0	8.3 ± 3.2	7.9 ± 3.3	0.1 ± 2.0	0.4 ± 1.9

Legend: M: Mean; SD: Standard Deviation; DRS: Dispositional Resilience Scale; PRE: Pre-COVID-19 period; POST: Post-COVID-19 period; Δ: difference between post- and pre-COVID-19 periods.

**Table 5:** Repeated measures ANOVA model of time (i.e., pre- and post-pandemic first-wave effect) and ward effects on DRS total. The model assesses the impact of the first wave of COVID-19 on total hardiness for nurses directly involved in caring for COVID-19 patients and on those who did not.

**Dependent Variables**	**COVID Ward**	**Average**	**SD**	**N**
DRS Total (Pre)	No	42.92	5.63	838
	Yes	42.98	5.25	412
DRS Total (Post)	No	41.91	6.63	838
	Yes	41.07	6.87	412
**Multivariate Test**				
**Effects**	**Wilks’ λ**	**F**	**Sig**	
Time	0.93	94.30	*p* < 0.001	
Time × COVID Ward	0.99	8.93	*p* = 0.003	

Note: × represents a combination of two variables. The same as below tables.

**Table 6:** Repeated measures ANOVA model of time (i.e., pre- and post-pandemic first-wave effect) and ward effects on DRS commitment. The model assesses the impact of the first wave of COVID-19 on the commitment subscale for nurses directly involved in caring for COVID-19 patients and on those who did not.

**Dependent Variables**	**COVID Ward**	**Average**	**SD**	**N**
DRS Commitment (Pre)	No	15.09	2.45	838
	Yes	15.11	2.34	412
DRS Commitment (Post)	No	14.34	3.03	838
	Yes	14.07	3.07	412
**Multivariate Test**				
**Effects**	**Wilks’ λ**	**F**	**Sig**	
Time	0.88	169.14	*p* < 0.001	
Time × COVID Ward	0.99	4.10	*p* = 0.043	

**Table 7:** Repeated measures ANOVA model of time (i.e., pre- and post-pandemic first-wave effect) and ward effects on DRS control. The model assesses the impact of the first wave of COVID-19 on the control subscale for nurses directly involved in caring for COVID-19 patients and on those who did not.

**Dependent Variables**	**COVID Ward**	**Average**	**SD**	**N**
DRS Control (Pre)	No	14.40	2.25	838
	Yes	14.50	2.06	412
DRS Control (Post)	No	14.24	2.42	838
	Yes	14.08	2.44	412
**Multivariate Test**				
**Effects**	**Wilks’ λ**	**F**	**Sig**	
Time	0.98	25.81	*p* < 0.001	
Time × COVID Ward	0.99	5.01	*p* = 0.025	

**Table 8:** Repeated measures ANOVA model of time (i.e., pre- and post-pandemic first-wave effect) and ward effects on DRS challenge. The model assesses the impact of the first wave of COVID-19 on resilience for nurses directly involved in caring for COVID-19 patients and on those who did not.

**Dependent Variables**	**COVID Ward**	**Average**	**SD**	**N**
DRS Challenge (Pre)	No	13.42	3.05	838
	Yes	13.36	3.02	412
DRS Challenge (Post)	No	13.34	3.18	838
	Yes	12.92	3.30	412
**Multivariate Test**				
**Effects**	**Wilks’ λ**	**F**	**Sig**	
Time	0.99	18.20	*p* < 0.001	
Time × COVID Ward	0.99	8.49	*p* = 0.004	

**Table 9:** Repeated measures ANOVA model of time (i.e., pre- and post-pandemic first-wave effect) and PPE effects on hardiness.

**Dependent Variables**	**PPE**	**Average**	**SD**	**N**
DRS Total (Pre)	No	43.17	5.41	200
	Yes	42.98	5.25	212
DRS Total (Post)	No	39.93	7.25	200
	Yes	42.15	6.31	212
**Multivariate Test**				
**Effects**	**Wilks’ λ**	**F**	**Sig**	
Time	0.87	58.83	*p* < 0.001	
Time × PPE	0.94	26.37	*p* < 0.001	

**Table 10:** Repeated measures ANOVA model of time (i.e., pre- and post-pandemic first-wave effect) and PPE effects on commitment hardiness.

**Dependent Variables**	**PPE**	**Average**	**SD**	**N**
DRS Commitment (Pre)	No	15.03	2.50	200
	Yes	15.18	2.20	212
DRS Commitment (Post)	No	13.42	3.21	200
	Yes	14.69	2.80	212
**Multivariate Test**				
**Effects**	**Wilks’ λ**	**F**	**Sig**	
Time	0.84	79.81	*p* < 0.001	
Time × PPE	0.95	22.47	*p* < 0.001	

**Table 11:** Repeated measures ANOVA model of time (i.e., pre- and post-pandemic first-wave effect) and PPE effects on control hardiness.

**Dependent Variables**	**PPE**	**Average**	**SD**	**N**
DRS Control (Pre)	No	14.68	2.20	200
	Yes	14.34	1.92	212
DRS Control (Post)	No	13.86	2.62	200
	Yes	14.29	2.25	212
**Multivariate Test**				
**Effects**	**Wilks’ λ**	**F**	**Sig**	
Time	0.95	22.20	*p* < 0.001	
Time × PPE	0.96	17.26	*p* < 0.001	

**Table 12:** Repeated measures ANOVA model of time (i.e., pre- and post-pandemic first-wave effect) and PPE effects on challenge hardiness.

**Dependent Variables**	**PPE**	**Average**	**SD**	**N**
DRS Challenge (Pre)	No	13.46	3.05	200
	Yes	13.27	2.99	212
DRS Challenge (Post)	No	12.66	3.37	200
	Yes	13.17	3.22	212
**Multivariate Test**				
**Effects**	**Wilks’ λ**	**F**	**Sig**	
Time	0.95	20.55	*p* < 0.001	
Time × PPE	0.97	12.79	*p* < 0.001	

**Table 13:** Statistical correlation between satisfaction levels about ward/department reallocation due to COVID-19 and DRS scale with 4 scores (post and delta description).

Resilience Dimension	Satisfaction	*p*-Value
Post DRS: Total	0.496	*p* < 0.001
Post DRS: Commitment	0.507	*p* < 0.001
Post DRS: Control	0.394	*p* < 0.001
Post DRS: Challenge	0.312	*p* < 0.001
Δ DRS: Total	0.498	*p* < 0.001
Δ DRS: Commitment	0.479	*p* < 0.001
Δ DRS: Control	0.466	*p* < 0.001
Δ DRS: Challenge	0.318	*p* < 0.001

Legend: DRS: Dispositional Resilience Scale; POST: Post-COVID-19 period; Δ: difference between post and pre.

**Table 14:** Repeated measures ANOVA model of time (i.e., pre- and post-pandemic first-wave effect) and geographical area effects on hardiness.

**Dependent Variables**	**Place**	**Average**	**SD**	**N**
DRS Total (Pre)	North	42.66	5.12	192
	Center	43.25	5.27	211
DRS Total (Post)	North	40.27	7.10	192
	Center	41.80	6.64	211
**Multivariate Test**				
**Effects**	**Wilks’ λ**	**F**	**Sig**	
Time	0.88	53.15	*p* < 0.001	
Time × geographical area	0.99	3.15	*p* = 0.077	

**Table 15:** Repeated measures ANOVA model of time (i.e., pre- and post-pandemic first-wave effect) and geographical area effects on commitment hardiness.

**Dependent Variables**	**Place**	**Average**	**SD**	**N**
DRS Commitment (Pre)	North	14.88	2.28	192
	Center	15.32	2.38	211
DRS Commitment (Post)	North	13.56	3.10	192
	Center	14.54	3.01	211
**Multivariate Test**				
**Effects**	**Wilks’ λ**	**F**	**Sig**	
Time	0.84	74.29	*p* < 0.001	
Time × geographical area	0.99	4.73	*p* = 0.030	

**Table 16:** Repeated measures ANOVA model of time (i.e., pre- and post-pandemic first-wave effect) and geographical area effects on control hardiness.

**Dependent Variables**	**Place**	**Average**	**SD**	**N**
DRS Control (Pre)	North	14.33	2.01	192
	Center	14.68	2.11	211
DRS Control (Post)	North	13.67	2.44	192
	Center	14.47	2.41	211
**Multivariate Test**				
**Effects**	**Wilks’ λ**	**F**	**Sig**	
Time	0.95	20.67	*p* < 0.001	
Time × geographical area	0.99	5.42	*p* = 0.020	

**Table 17:** Repeated measures ANOVA model of time (i.e., pre- and post-pandemic first-wave effect) and geographical area effects on challenge hardiness.

**Dependent Variables**	**Place**	**Average**	**SD**	**N**
DRS Challenge (Pre)	North	13.46	3.01	192
	Center	13.25	2.99	211
DRS Challenge (Post)	North	13.05	3.31	192
	Center	12.79	3.31	211
**Multivariate Test**				
**Effects**	**Wilks’ λ**	**F**	**Sig**	
Time	0.96	18.49	*p* < 0.001	
Time × geographical area	1.00	0.05	*p* = 0.830	

**Table 18:** Correlation between state anxiety and the DRS scale of nurses caring for COVID-19 patients.

DRS Dimensions	State Anxiety	*p*-Value
Post DRS: Total	−0.618	*p* < 0.001
Post DRS: Commitment	−0.536	*p* < 0.001
Post DRS: Control	−0.410	*p* < 0.001
Post DRS: Challenge	−0.487	*p* < 0.001
Δ DRS: Total	−0.477	*p* < 0.001
Δ DRS: Commitment	−0.449	*p* < 0.001
Δ DRS: Control	−0.377	*p* < 0.001
Δ DRS: Challenge	−0.344	*p* < 0.001

Legend: DRS: Dispositional Resilience Scale; POST: Post-COVID-19 period; Δ: difference between post-COVID-19 and pre-COVID-19 periods.

**Table 19:** Hardiness predictors according to multivariate analysis (service seniority, transfer evaluation, length of service × anxiety).

Best Model Goodness of Fit: Δ DRS Total			
*x* ^2^	df	Likelihood logarithm	Nagelkerke R^2^
39.12 ***	3	115.82	0.393
Variable	B	Wald	Exp(B)
Length of service	0.196	11.95 ***	1.217
Satisfaction levels about ward/department reallocation	0.775	12.39 ***	2.170
Length of service × Anxiety	−0.003	8.15 ***	0.997
Percentage of correct classification	Δ DRS Total < 0		74.6%
	Δ DRS Total ≥ 0		66%

***: *p* < 0.001. Legend: *x*^2^: chi-square; df: Degree of Freedom; B: unstandardized beta; Wald: Wald test; Exp(B); exponentiation of the B coefficient.

**Table 20:** Commitment predictors according to multivariate analysis (length of service, transfer evaluation, length of service × anxiety, and insufficient PPE (no) × transfer evaluation) and correct/incorrect classification of the model.

Best Model Goodness of Fit: Δ DRS Commitment			
*x* ^2^	Df	Likelihood logarithm	Nagelkerke R^2^
46.67 ***	4	108.59	0.454
Variable	B	Wald	Exp(B)
Length of service	0.204	11.03 ***	1.226
Satisfaction levels about ward/department reallocation	0.674	9.14 ***	1.963
Length of service × Anxiety	−0.004	8.74 ***	0.996
Insufficient PPE × Satisfaction levels about ward/department reallocation	0.385	8.29 ***	1.469
Percentage of correct classification	Δ DRS Commitment < 0		82.1%
	Δ DRS Commitment ≥ 0		71.4%

***: *p* < 0.001. Legend: *x*^2^: chi-square; df: Degree of Freedom; B: unstandardized beta; Wald: Wald test; Exp(B); exponentiation of the B coefficient.

**Table 21:** Control predictors according to multivariate analysis (transfer evaluation, anxiety × transfer evaluation) and correct/incorrect classification of the model.

Best Model Goodness of Fit: Δ DRS Control			
*x* ^2^	Df	Likelihood logarithm	Nagelkerke R^2^
24.44 ***	2	125.64	0.266
Variable	B	Wald	Exp(B)
Satisfaction levels about ward/department reallocation	1.418	17.03 ***	4.127
Anxiety × Satisfaction levels about ward/department reallocation	−0.017	9.07 ***	0.983
Percentage of correct classification	Δ DRS Control < 0		52.3%
	Δ DRS Control ≥0		83.8%

***: *p* < 0.001. Legend: *x*^2^: chi-square; df: Degree of Freedom; B: unstandardized beta; Wald: Wald test; Exp(B); exponentiation of the B coefficient.

**Table 22:** Challenge predictors according to multivariate analysis (anxiety, anxiety × seniority of service, anxiety × transfer evaluation, PPE (no) × transfer evaluation) and correct/incorrect classification of the model.

Best Model Goodness of Fit: Δ DRS Challenge			
*x* ^2^	df	Likelihood logarithm	Nagelkerke R^2^
38.38 ***	4	105.10	0.402
Variable	B	Wald	Exp(B)
Anxiety	−0.06	5.76 **	0.942
Anxiety × Length of service	−0.002	5.15 *	0.998
Length of service × Satisfaction levels about ward/department reallocation	0.36	8.58 ***	1.037
Insufficient PPE × Satisfaction levels about ward/department reallocation	−1.135	7.96 ***	0.322
Percentage of correct classification	Δ DRS Challenge < 0		44.7%
	Δ DRS Challenge ≥ 0		89.2%

*: *p* < 0.05; **: *p* < 0.01; ***: *p* < 0.001. Legend: *x*^2^: chi-square; df: Degree of Freedom; B: unstandardized beta; Wald: Wald test; Exp(B); exponentiation of the B coefficient.

## Data Availability

The data presented in this study are available on request from the corresponding author.

## References

[1] Mendelson M., Nel J., Blumberg L., Madhi S.A., Dryden M., Stevens W., Venter F.W.D. (2020). Long-COVID: An evolving problem with an extensive impact. S. Afr. Med. J..

[2] Xiong J., Lipsitz O., Nasri F., Lui L.M.W., Gill H., Phan L., Chen-Li D., Iacobucci M., Ho R., Majeed A. (2020). Impact of COVID-19 pandemic on mental health in the general population: A systematic review. J. Affect. Disord..

[3] Wu T., Jia X., Shi H., Niu J., Yin X., Xie J., Wang X. (2021). Prevalence of mental health problems during the COVID-19 pandemic: A sys-tematic review and meta-analysis. J. Affect. Disord..

[4] Varghese A., George G., Kondaguli S.V., Naser A.Y., Khakha D.C., Chatterji R. (2021). Decline in the mental health of nurses across the globe during COVID-19: A systematic review and meta-analysis. J. Glob. Health.

[5] Preti E., Di Mattei V., Perego G., Ferrari F., Mazzetti M., Taranto P., Di Pierro R., Madeddu F., Calati R. (2020). The psychological impact of epidemic and pandemic outbreaks on healthcare workers: Rapid review of the evidence. Curr. Psychiatry Rep..

[6] Cabarkapa S., Nadjidai S.E., Murgier J., Ng C.H. (2020). The psychological impact of COVID-19 and other viral epidemics on frontline healthcare workers and ways to address it: A rapid systematic review. Brain Behav. Immun. Health.

[7] Epstein R.M., Krasner M.S. (2013). Physician resilience: What it means, why it matters, and how to promote it. Acad. Med..

[8] Carmassi C., Foghi C., Dell’Oste V., Cordone A., Bertelloni C.A., Bui E., Dell’Osso L. (2020). PTSD symptoms in healthcare workers facing the three coronavirus outbreaks: What can we expect after the COVID-19 pandemic. Psychiatry Res..

[9] Baskin R.G., Bartlett R. (2021). Healthcare worker resilience during the COVID-19 pandemic: An integrative review. J. Nurs. Manag..

[10] Labrague L.J. (2021). Psychological resilience, coping behaviours and social support among health care workers during the COVID-19 pandemic: A systematic review of quantitative studies. J. Nurs. Manag..

[11] Luthans F., Youssef-Morgan C.M. (2017). Psychological capital: An evidence-based positive approach. Annu. Rev. Organ. Psychol. Organ. Behav..

[12] Hu T., Zhang D., Wang J. (2015). A meta-analysis of the trait resilience and mental health. Personal. Individ. Differ..

[13] Picardi A., Bartone P.T., Querci R., Bitetti D., Tarsitani L., Roselli V., Maraone A., Fabi E., De Michele F., Gaviano I. (2012). Development and validation of the Italian version of the 15-item dispositional resilience scale. Riv. Psichiatr..

[14] Bartone P.T. (2007). Test-retest reliability of the dispositional resilience scale-15, a brief hardiness scale. Psychol. Rep..

[15] Solano J.P., Bracher E.S., Faisal-Cury A., Ashmawi H.A., Carmona M.J., Lotufo F.N., Vieira J.E. (2016). Factor structure and psychometric properties of the Dispositional Resilience Scale among Brazilian adult patients. Arq. Neuropsiquiatr..

[16] Maddi S.R. (2002). The story of hardiness: Twenty Years of theorizing, research, and practice. Consult. Psychol. J.: Pract. Res..

[17] Kobasa S.C., Maddi S.R., Puccetti M.C. (1982). Personality and exercise as buffers in the stress-illness relationship. J. Behav. Med..

[18] Daly L.M. (2020). Resilience: An integrated review. Nurs. Sci. Q..

[19] Chen S.X., Lopez S.J. (2009). Hardiness. The Encyclopedia of Positive Psychology.

[20] Prati G. (2010). Proprietà psicometriche della scala della resilienza disposizionale. G. Psicol..

[21] Eschleman K.J., Bowling N.A., Alarcon G.M. (2010). A meta-analytic examination of hardiness. Int. J. Stress Manag..

[22] Dolbier C.L., Smith S.E., Steinhardt M.A. (2007). Relationships of protective factors to stress and symptoms of illness. Am. J. Health Behav..

[23] Gito M., Ihara H., Ogata H. (2013). The relationship of resilience, hardiness, depression and burnout among Japanese psychiatric hospital nurses. J. Nurs. Educ. Pract..

[24] Park J.S., Lee E.H., Park N.R., Choi Y.H. (2018). Mental health of nurses working at a government-designated hospital during a MERS-CoV outbreak: A cross-sectional study. Arch. Psychiatr. Nurs..

[25] Maiorano T., Vagni M., Giostra V., Pajardi D. (2020). COVID-19: Risk factors and protective role of resilience and coping strategies for emergency stress and secondary trauma in medical staff and emergency workers—An online-based inquiry. Sustainability.

[26] Vagni M., Maiorano T., Giostra V., Pajardi D. (2020). Hardiness, stress and secondary trauma in Italian healthcare and emergency workers during the COVID-19 pandemic. Sustainability.

[27] Pedrabissi L., Santinello M. (1989). Verifica della validità dello STAI forma Y di Spielberger [Verification of the validity of the STAI, Form Y, by Spielberger]. Giunti Organ. Spec..

[28] Wong J.Y., Fong D.Y., Choi A.W., Chan C.K., Tiwari A., Chan K.L., Lai V., Logan T., Bartone P. (2014). Transcultural and psychometric validation of the Dispositional Resilience Scale (DRS-15) in Chinese adult women. Qual. Life Res..

[29] Spielberger C.D. (1983). State-Trait Anxiety Inventory.

[30] Ramanaiah N.V., Franzen M., Schill T. (1983). A psychometric study of the State-Trait Anxiety Inventory. J. Pers. Assess..

[31] IBM Corp (2020). IBM SPSS Statistics for Macintosh, Version 27.0.

[32] (2020). Direzione Generale delle Professioni Sanitarie e delle Risorse Umane del SSN –Ufficio III “Il Personale del Sistema Sanitario Italiano. Anno 2018”. https://www.salute.gov.it/imgs/C_17_pubblicazioni_3011_allegato.pdf.

[33] Bosa I., Castelli A., Castelli M., Ciani O., Compagni A., Galizzi M.M., Garofano M., Ghislandi S., Giannoni M., Marini G. (2021). Response to COVID-19: Was Italy (un)prepared?. Health Econ. Policy Law.

[34] Bambi S., Iozzo P., Lucchini A. (2020). New Issues in Nursing Management During the COVID-19 Pandemic in Italy. Am. J. Crit. Care.

[35] Hoogendoorn M.E., Brinkman S., Bosman R.J., Haringman J., de Keizer N.F., Spijkstra J.J. (2021). The impact of COVID-19 on nursing work-load and planning of nursing staff on the Intensive Care: A prospective descriptive multicenter study. Int. J. Nurs. Stud..

[36] Lucchini A., Giani M., Elli S., Villa S., Rona R., Foti G. (2020). Nursing Activities Score is increased in COVID-19 patients. Intensive Crit. Care Nurs..

[37] González-Gil M.T., González-Blázquez C., Parro-Moreno A.I., Pedraz-Marcos A., Palmar-Santos A., Otero-García L., Navar-ta-Sánchez M.V., Alcolea-Cosín M.T., Argüello-López M.T., Canalejas-Pérez C. (2021). Nurses’ perceptions and demands regarding COVID-19 care delivery in crit-ical care units and hospital emergency services. Intensive Crit. Care Nurs..

[38] Heesakkers H., Zegers M., van Mol M.M.C., van den Boogaard M. (2021). The impact of the first COVID-19 surge on the mental well-being of ICU nurses: A nationwide survey study. Intensive Crit. Care Nurs..

[39] Setti I., Argentero P. (2012). Vicarious trauma: A contribution to the Italian adaptation of the Secondary Traumatic Stress Scale in a sample of ambulance operators. Appl. Psychon. Bull..

[40] Jose S., Dhandapani M., Cyriac M.C. (2020). Burnout and Resilience among Frontline Nurses during COVID-19 Pandemic: A Cross-sectional Study in the Emergency Department of a Tertiary Care Center, North India. Indian J. Crit. Care Med..

[41] Maddi S.R. (2007). Relevance of hardiness assessment and training to the military context. Mil. Psychol..

[42] Judkins J.L., Moore B.A., Collette T. (2020). Psychological Hardiness. The Routledge Research Encyclopedia of Psychology Applied to Every-Day Life.

[43] Saglietto A., D’Ascenzo F., Zoccai G.B., De Ferrari G.M. (2020). COVID-19 in Europe: The Italian lesson. Lancet.

[44] Lasalvia A., Bonetto C., Porru S., Carta A., Tardivo S., Bovo C., Ruggeri M., Amaddeo F. (2020). Psychological impact of COVID-19 pandemic on healthcare workers in a highly burdened area of north-east Italy. Epidemiol. Psychiatr. Sci..

[45] Rodriguez R.M., Medak A.J., Baumann B.M., Lim S., Chinnock B., Frazier R., Cooper R.J. (2020). Academic emergency medicine physicians’ anxiety levels, stressors, and potential stress mitigation measures during the acceleration phase of the COVID-19 pandemic. Acad. Emerg. Med..

[46] Saran S., Gurjar M., Baronia A.K., Lohiya A., Azim A., Poddar B., Rao N.S. (2020). Personal protective equipment during COVID-19 pandemic: A narrative review on technical aspects. Expert Rev. Med. Devices.

[47] Azizpour I., Mehri S., Moghaddam H.R., Mirzaei A., Soola A.H. (2021). The impact of psychological factors on bereavement among frontline nurses fighting Covid-19. Int. J. Afr. Nurs. Sci..

[48] Bergua V., Meillon C., Potvin O., Bouisson J., Le Goff M., Rouaud O., Ritchie K., Dartigues J.F., Amieva H. (2012). The STAI-Y trait scale: Psycho-metric properties and normative data from a large population-based study of elderly people. Int. Psychogeriatr..

